# Supplementary figures and images for: Metabolism fine tuning and cardiokines secretion represent adaptative responses of the heart to High Fat and High Sugar Diets in flies
Source: PLoS Genet. 2026 Jun 11;22(6):e1012189. doi: 10.1371/journal.pgen.1012189 (PMC13286269; doi:10.1371/journal.pgen.1012189)

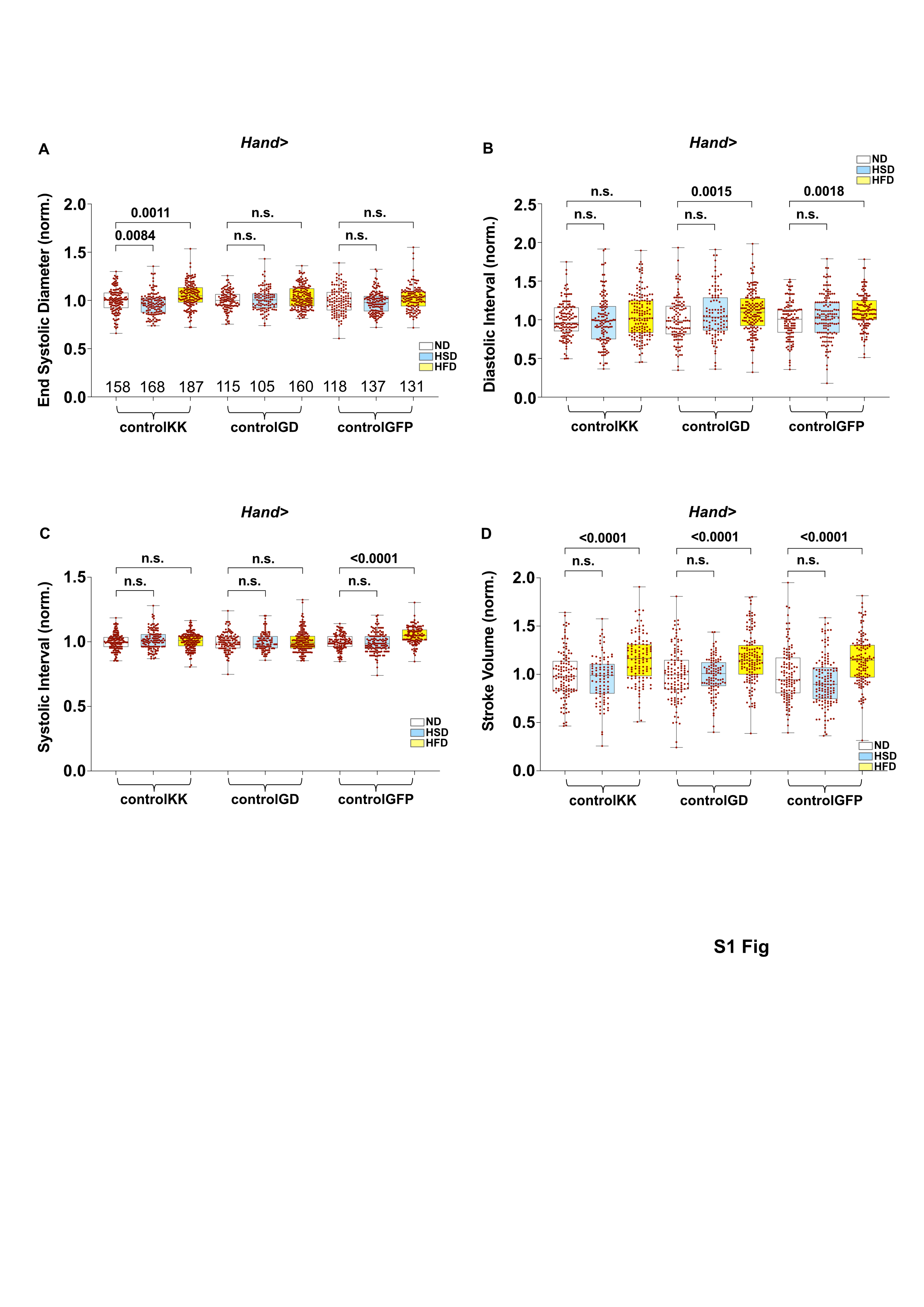

Supplement: S1 Fig — Measurements of cardiac parameters in the 3 types of Hand>control flies used in this study following 10days in ND and HSD or 3 days in HFD. Box plots showing normalized values for ESD (A), DI (B), SI (C) and SV (D). ND, white plots; HSD, blue plots; HFD, yellow plots. Numbers corresponds to the individuals co-evaluated in each diet. Statistical significance was tested using Kruskal-Wallis with Dunn’s multiple comparisons test. Significant p-values are indicated. (TIF) [file pgen.1012189.s001.tif]

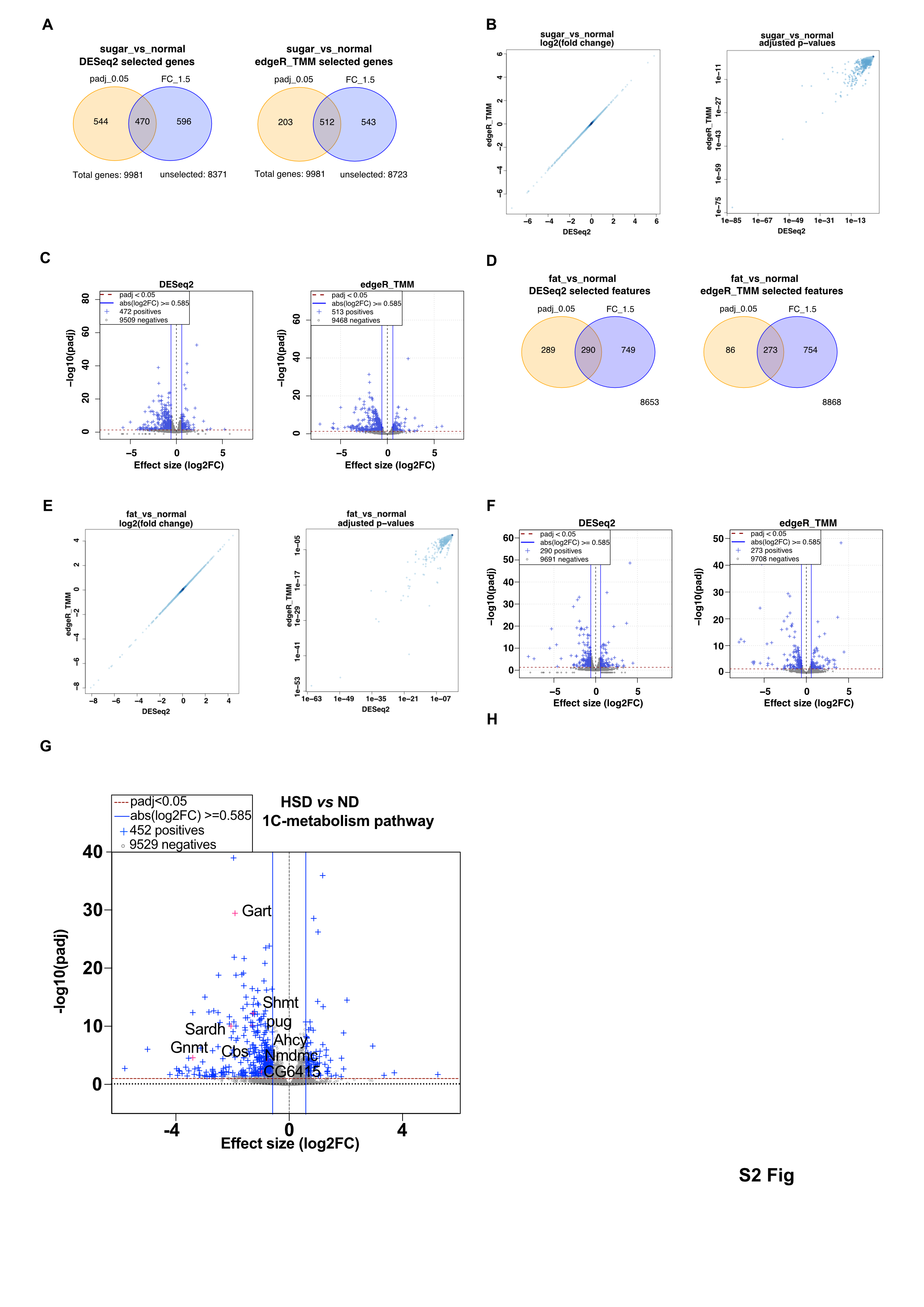

Supplement: S2 Fig — (A-C) Compared analysis for HSD versus ND results. (D-F) Compared analysis for HFD versus ND results. (A,D) Venn diagrams of selected genes (padj. < 0.05, FC>=1.5). After filtering, 9981 genes were analyzed with each method. Sugar or Fat hits in yellow and normal genes in blue. (B,E) Comparison of genes according to log2FC (left panels) or padj. value (right panels) with each method. (C,F) Volcano plots showing the repartition of the total genes in each condition determined in DEseq2 versus edgeR_TMM. padj<0.05, abs(log2FC)>=0.585, blue crosses indicate significant hits versus grey n.s. (G) Volcano plot highlighting members of the 1C-metabolism pathway downregulated in HSD versus ND condition. (TIF) [file pgen.1012189.s002.tif]

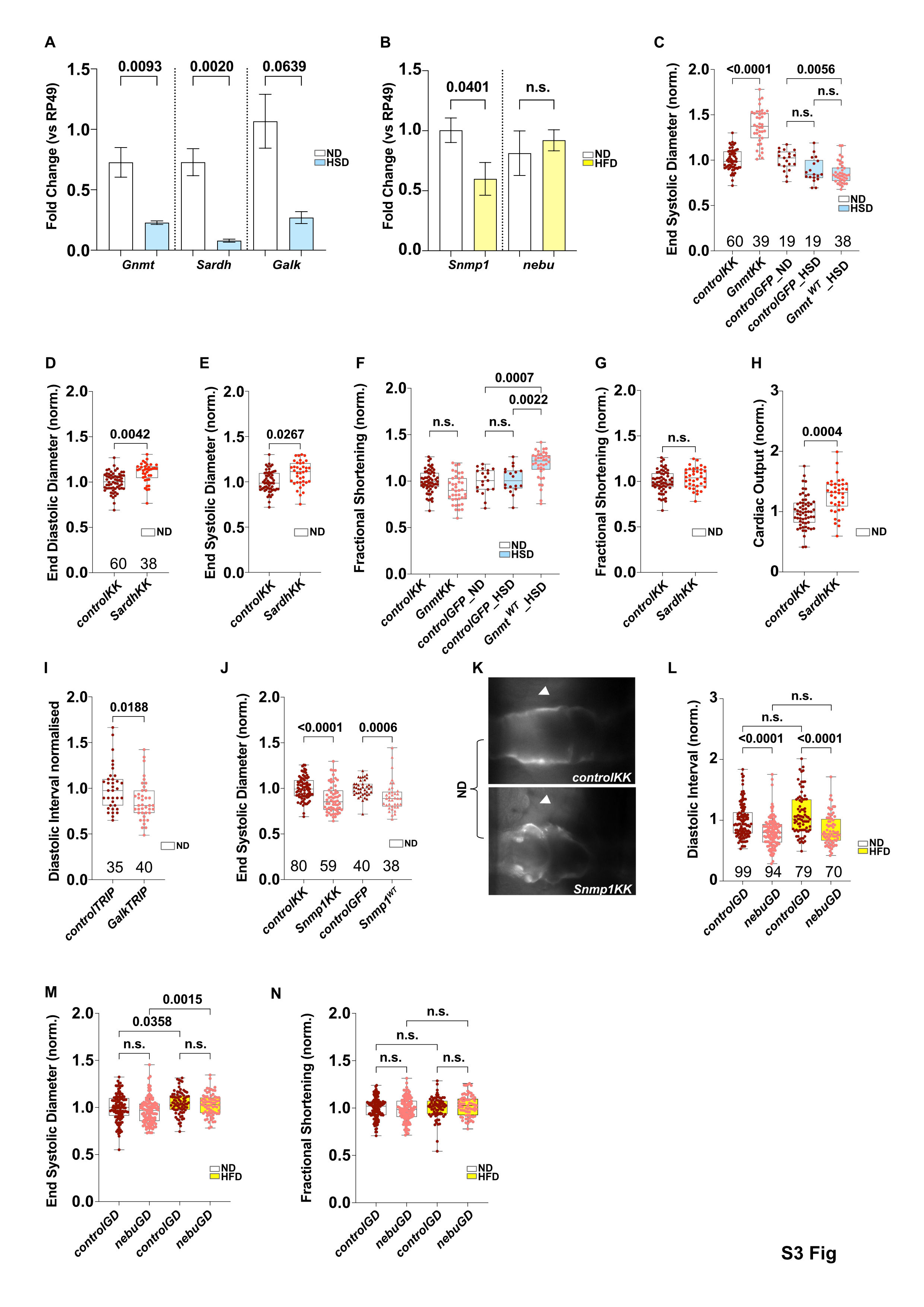

Supplement: S3 Fig — Fold Changes are normalized to RP49. Statistical significance was tested unpaired t-test with Welch’s correction. (C) Effect on ESD of Gnmt knockdown in ND (white box plots) and of Gnmt overexpression in HSD (blue box plots) compared to respective controls. (D-E) Effect of driven Sardh knockdown (SardhKK) on EDD (E) and ESD (F) compared to>controlKK in ND. (F) Effect on FS of Gnmt knockdown in ND (white box plots) and of Gnmt overexpression in HSD (blue box plots) compared to respective controls. (G-H) Effect of Sardh knockdown (SardhKK) on FS (E) and CO (F) compared to>controlKK in ND. (I) Effect of GalK knockdown (GalkTRIP) on DI compared to control in ND. (J) Effect of Snmp1 knockdown (Snmp1KK) and overexpression (Snmp1WT) on ESD compared to respective controls in ND. (K) Image capture from representative movies showing abdominal fat accumulation (arrowhead) in Hand> driven Snmp1KK and control. (L-O) Effect of nebu knockdown (nebuGD) on DI (L), ESD (M), FS (N) and AI (O) compared to controls in ND (white box plots) and in HFD (yellow box plots). Crosses are performed with Hand > . Phenotypic values of tested conditions where normalized to corresponding controls. Numbers of individual flies co-evaluated in each diet are presented. Statistical significance was tested using Kruskal-Wallis with Dunn’s multiple comparisons test. p-values are indicated. (TIF) [file pgen.1012189.s003.tif]

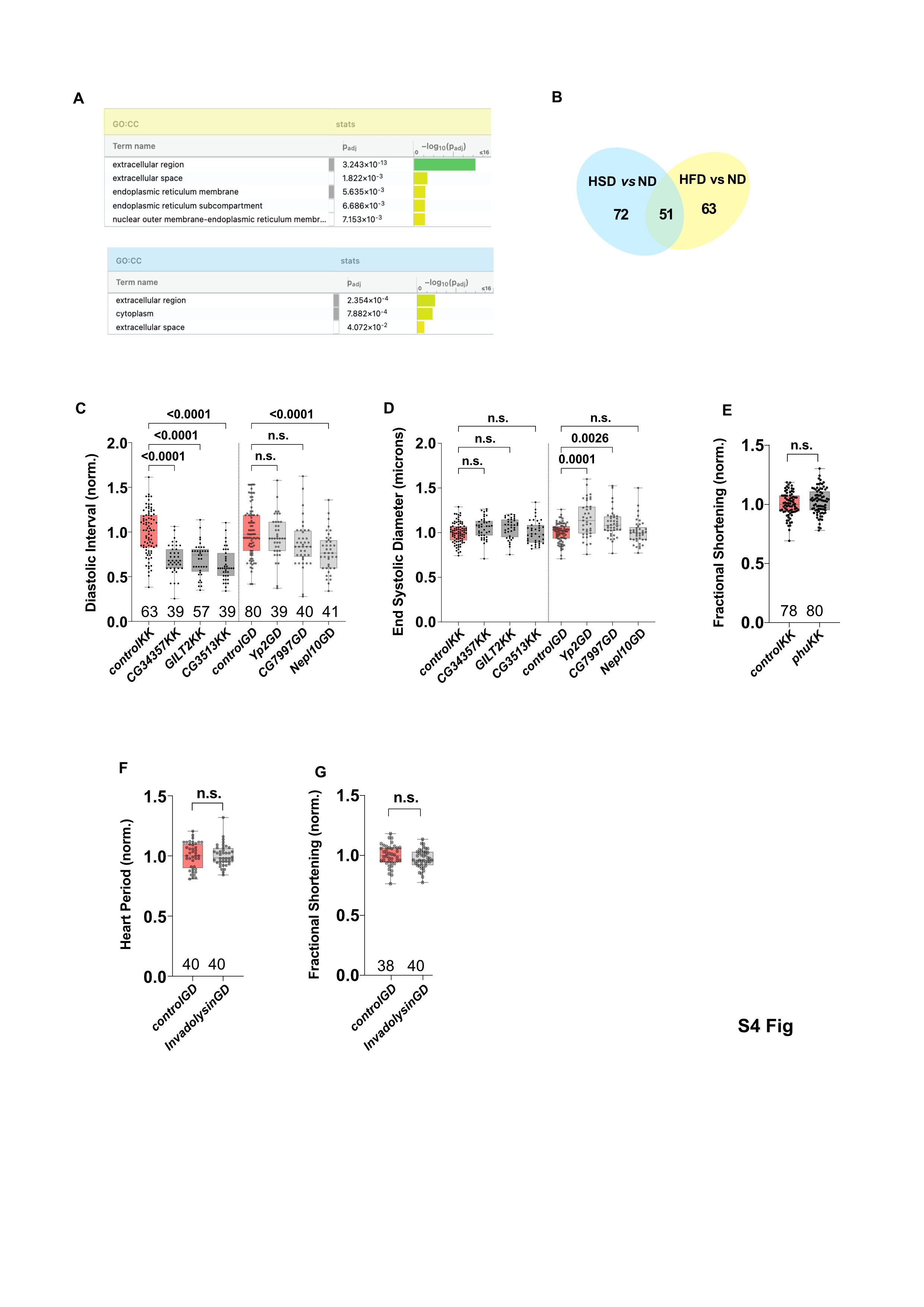

Supplement: S4 Fig — (B) Venn diagram of DEG identified with Biomart/SignalP 5.0 in HSD and HFD versus ND (padj<0.05). (C) DI and (D) ESD modifications in Hand> driven KD (grey) compared to respective controls (red). (E) Effect of Hand> driven Invadolysin KD on HP and (F) FS compared to control. Values where normalized to controls. The number of individuals analyzed in each condition (genotype/diet) is indicated. Statistical significance was tested using Kruskal-Wallis with Dunn’s multiple comparisons test. Significant p-values are indicated. Genotypes: UAS-CG34357KK, UAS-GILT2KK, UAS-CG3513KK, UAS-Yp2GD, UAS-CG7997GD, UAS-Nepl10, UAS-InvadolysinGD. (TIF) [file pgen.1012189.s004.tif]

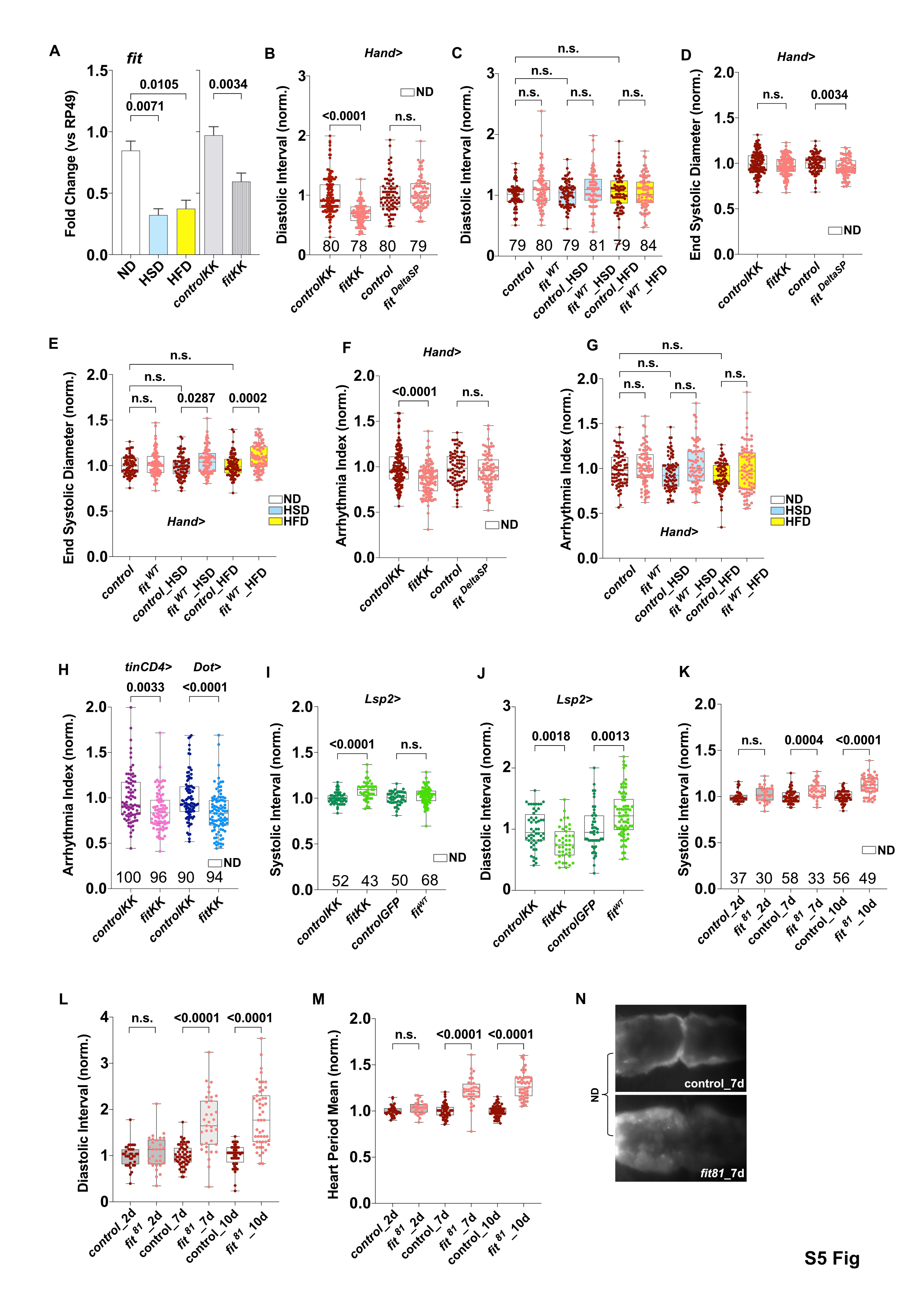

Supplement: S5 Fig — Statistical significance was tested unpaired t-test with Welch’s correction. (B-C) Effect on DI of fitKK and fitDeltaSP in ND (B) and of fitWT in ND, HSD, HFD (C) compared to respective controls. (D-E) Effect on ESD of fitKK and fitDeltaSP in ND (D) and of fitWT in ND, HSD, HFD (E) compared to respective controls. (F-G) Effect on AI of fitKK and fitDeltaSP in ND (F) and of fitWT in ND, HSD, HFD (G) compared to respective controls. (H) Effect on AI of fit knockdown (fitKK) in cardiomyocytes (B, tinCD4>) and in pericardial cells (C, Dot>) compared to respective control flies in ND. (I-J) Effect of fitKK and fitWT in ND on SI (I) and DI (J) when expressed in fat body (Lsp2>) compared to respective controls. (K-M) Effect of mutant fit81 in females at 2, 7 and 10 days old, on SI (K), DI (L) and HP (M) compared to controls. (N) Image capture from representative movies showing thickening of the heart wall and obstructed heart lumen in fit81 mutant females compared to control at 7 days-old. Except when mentioned, all crosses were performed with Hand > . Phenotypic values of tested conditions where normalized to corresponding controls. Numbers of individual flies co-evaluated in each diet are presented. Statistical significance was tested using Kruskal-Wallis with Dunn’s multiple comparisons test. Significant p-values are indicated. (TIF) [file pgen.1012189.s005.tif]

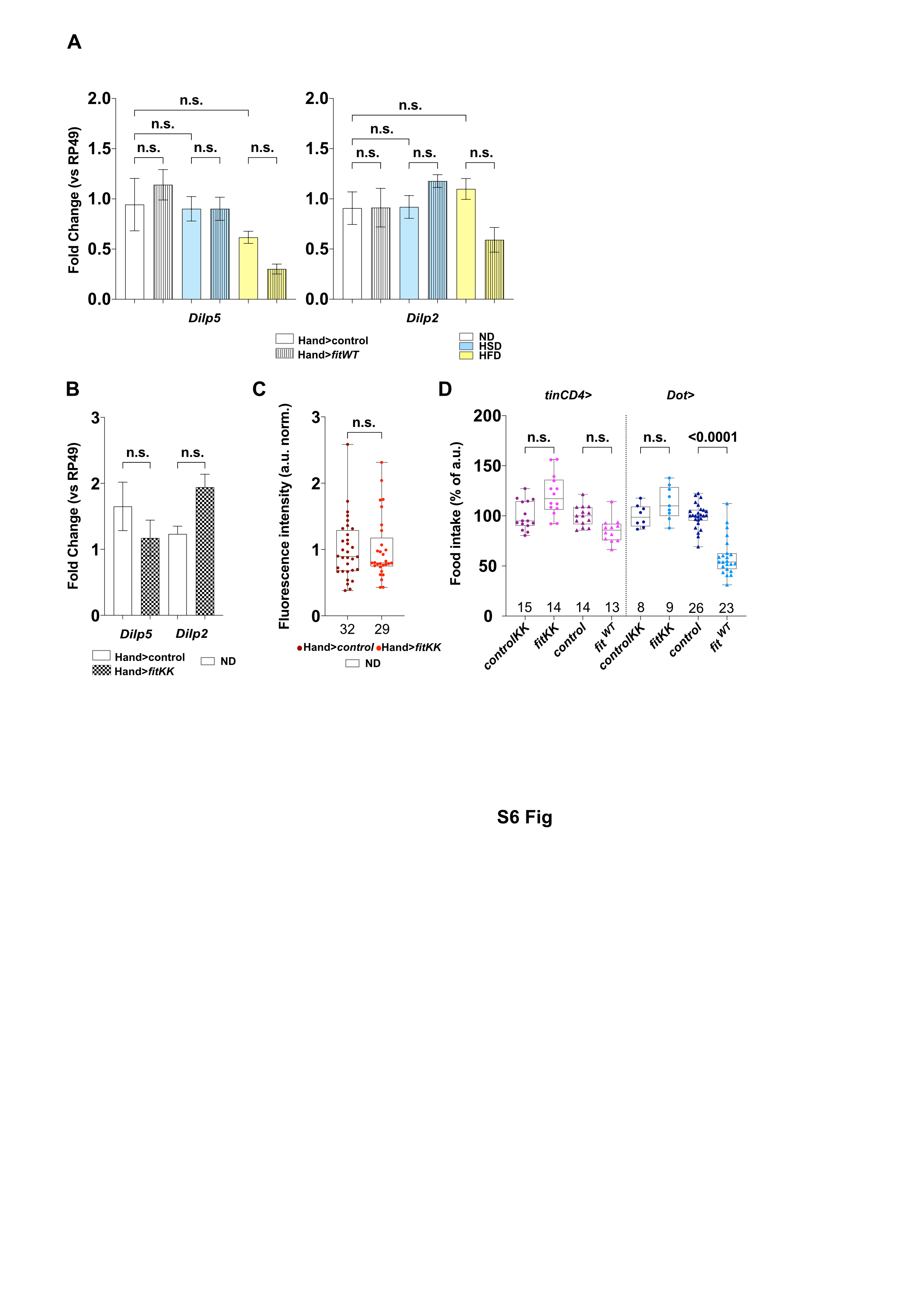

Supplement: S6 Fig — Fold Changes are normalized to RP49. Statistical significance was tested unpaired t-test with Welch’s correction. (C) Fluorescence intensity of Dilp5 immunostaining measured in IPCs from dissected adult female brains fed ND. Genotypes: Hand>fitKK and Hand>control. Values are normalized to controls. Statistical significance was tested using Kruskal-Wallis with Dunn’s multiple comparisons test. (D) Quantification of food intake tinCD4> or Dot> driven fit knockdown (fitKK) and overexpression (fitWT) females starved overnight and refed 3 hours. Values are normalized to respective controls. Numbers corresponds to averaged values for groups of 10 adults obtained from 3-6 independent experiments. Statistical significance was tested using Kruskal-Wallis with Dunn’s multiple comparisons test. (TIF) [file pgen.1012189.s006.tif]
