# Supplementary material for: Metabolism fine tuning and cardiokines secretion represent adaptative responses of the heart to High Fat and High Sugar Diets in flies
Source: PLoS Genet. 2026 Jun 11;22(6):e1012189. doi: 10.1371/journal.pgen.1012189 (PMC13286269; doi:10.1371/journal.pgen.1012189)
Supplement: Attachment — Submitted filename: 152348_1_rebuttal_3721507_td65ph.pdf [file pgen.1012189.s012.pdf]

Full Revision

Manuscript number: RC-2024-02774
Corresponding author(s): Nathalie Arquier

1. General Statements [optional]

Diabetic cardiomyopathies are one of the leading causes of death in obese diabetics. The etiology of these specific cardiac dysfunctions remains poorly understood due to the multiple causes of the disease onset. Damages induced results from a pleiotropy of physiological dysregulations affecting the stiffness and functioning of the cardiomyocytes.

Here, we used the valuable Drosophila model to mimic the onset of cardiac phenotypes associated to early molecular dysregulations in the cardiac cells. We identified genes encoding core components of key homeostatic pathways as well as a panel of genes encoding secreted proteins to be involved in the response of the heart to unhealthy diets. Our analysis notably identified and characterized the Fit hormone as a cardiokine in flies, which autonomously modulates the cardiac function and remotely affects feeding behavior.

Thanks to constructive comments and suggestions made the two referees, we strengthened our results by increasing samples, reanalyzing data and performing mandatory new experiments that are now included in this revised version.

Important: Please note reviewers token for Genome Omnibus access to GSE235313 data generated in this study: spermmawrfwnjyr

----
To review GEO accession GSE235313:
Go to https://www.ncbi.nlm.nih.gov/geo/query/acc.cgi?acc=GSE235313
Enter token spermmawrfwnjyr into the box
----

2. Point-by-point description of the revisions

Reviewer #1 (Evidence, reproducibility and clarity (Required)):

In this study, the authors investigated the effect of nutritional stress (HSD and HFD) on cardiac function by assessing multiple parameters on adult flies. They next identified the adaptive transcriptomic changes in the heart in response to these nutritional stresses and screened for their roles under ND, HSD and HFD. They identified fit gene, encoding a satiety gene, expressed by cardiomyocytes and pericardial cells.

I think the characterisation is thorough; however, the conclusion is not well supported by the evidence. My main concern is that in many graphs, the difference between control and experiment is subtle, and, secondly, the authors showed some conflicting results (e.g. one RNAi showed a reduction of one parameter, however, the other

independent RNAi did not. In this case, I believe the authors shouldn't conclude that the RNAi is functionally required, since the RNAis are meant to confirm each other.

First, we thank the reviewer for her/his constructive comments and suggestions. We obtained new results presented in the last version of the manuscript, which consistently support our conclusions and improve the study.

High-Sugar and High-Fat Diets modified cardiac performance

They assessed how HSD and HFD affect Adult fly heart performance. Instead of performing 3 weeks of dietary manipulation as has been done before by other groups, they put adult flies on HSD for 7 days and HFD for only 3 days.

We would like to clarify the nutritional challenge used. Cardiac function of flies was assessed at 10 days after emergence. Flies were put either in ND or HSD during these 10 days (ND and HSD conditions), or in ND for 7 days then transferred on HFD for 3days (HFD condition). Finally, all the females spent 10 days in a diet before being imaged or before hearts/brains dissection.

They found:
HSD increases HP and SI, and reduces AI. The difference is too small and not consistent between different control lines. Also, when the difference is this small, p value does not tell much!

They probably intentionally induced a milder effect so that they could assess adaptive transcriptomic changes to this nutritional stress. In Fig. 1D SI is increased under HSD with control-KK, In Fig. S1C, SI is not changed under HSD with control-GD and control-GFP. Instead, DI is increased, which is also opposite to what they showed in Fig. 1 C. HFD increased ESD, EDD, SV, FS and CO.(Hypertrophy). This is not true with control-GD and control-GFP lines though!
Comments: They have assessed many parameters in live animals with many different control lines, which is thorough. However, it is hard to draw any conclusions based on these conflicting results. Are these effect KK line specific?

Globally, we agree with the reviewer that the results, presented in the first version of the manuscript, for the control lines were difficult to understand due to the inconsistency of the phenotypes. In this revised version, we performed new results in **Figure 1** and **S1** regarding the effect of 10 days HSD and 3 days HFD exposure vs ND.
105 to 187 flies were imaged for the 3 control conditions, in the 3 diets concomitantly, to increase the power of our analysis. As mentioned in the main text (**page 3, line 30-35; page 4, line 1-5**), both diets deteriorate cardiac function with HFD leading to consistent phenotypes on heart diameters and rhythm and HSD milder effects. Indeed, the 3 control lines were uniformly affected by HFD after 3 days exposure, whereas 10 days in HSD was not sufficient to quantify a significant effect despite consistent the trends on several phenotypes (EDD, ESD, DI, AI and CO. These results revealed a different sensitivity of the cardiac performance when exposed to sugar and fat.
As described in the text, we were nevertheless confident that our approach would be good to investigate the early molecular dysregulations induced by sugar. This was the purpose of our analysis, presented in the follow-up of the manuscript.

Regarding the small differences measured in the phenotypes in HSD and HFD compared to ND, we would like to outline that the values presented are normalized values to control. Normalization is done for every independent experiment, performed at different dates, and permits the graphical representation of pooled values. Statistical analysis is performed using non-parametric Kruskal-Wallis test accordingly. Values are presented with the X axis cutting the Y axis at 0, this graphical representation also contributes to flattening the differences and p-values indicate their significance.

Analysis of the fly cardiac transcriptome upon nutritional stress

RNA seq to detect differentially expressed genes under HSD and HFD vs ND. Most DE genes are downregulated, which prompts them to assess how the downregulation of these genes adapts the animals to this nutritional stress.

High Sugar Diet downregulated 1c-metabolism and Leloir galactose pathways.

In this revised manuscript, we first present RT-qPCR validating the downregulation of *Gnmt*, *Sardh* and *Galk* expressions in the heart of 10days old HSD-fed females compared to ND-fed ones (**Figure S3A**).
We apologize for the confused explanations in the first version of the manuscript. We show new results in **Figure 3** and **S3** on the cardiac function of both *Gnmt* and *Sardh*, where following reviewer's suggestion, both genes were knocked down in the heart in ND and *Gnmt* overexpressed in HSD. No available tools allowed us to test *Sardh* overexpression in HSD and we could not get some for *Galk*.

GNMT is downregulated under HSD and HFD.

In ND, GNMT knockdown increased ESD, EDD and CO. Sardh knockdown did the same? However, Sardh knockdown did not affect ESD significantly.

We reanalyze our first data and added new ones, comparing only knockdown or overexpression to the corresponding controls performed in concomitant experiments. Results are now shown in **Figure 3C-E; S3C-H**. Knocking down *Gnmt* in the heart increased HP, EDD, ESD and CO, *Sardh* knockdown in ND resulted in milder phenotypes but inducing significant hypertrophy in ND as *Gnmt* does. In both cases, FS was not impacted.
Both genes have been previously shown as beneficial to muscular function in time-restricted feeding context (Livelo et al., 2023, Nat.Comm.), illustrating that, even if both enzymes are involved in opposite reaction, their function has the same effect on organ/tissue function, as they did for heart diameters. The text corresponding to results and discussion were updated accordingly **(pages 5, 11**).

The conclusion here is: GNMT knockdown induces hypertrophy, similar to the effect of HFD.

In HSD, further knockdown of GNMT reduced (rescued) HP, suggesting downregulation of GNMT under HSD is adaptive. Should overexpress GNMT under HSD to see if this manipulation further increases HP, to claim GNMT downregulation is an adaptive change to high sugar stress.

We thank the reviewer for her/his suggestion. We now used UAS-*GnmtWT* (from FlyORF) to assess the role of *Gnmt* on cardiac function in HSD.
As shown in (**Figure 3C-E; S3C,F**), overexpressing *Gnmt* in the heart in HSD was sufficient to rescue some sugar induced phenotypes or to induce other dysfunctions, when compared to corresponding controls evaluated in the same experiments in ND and HSD. Notably, HP increase and CO decrease are rescued by *Gnmt* cardiac overexpression in HSD. Interestingly, the cardiac diastolic constriction induced by HSD is associated to increased FS and CO in this genotype in sugar diet. These new results strengthen the positive effect of *Gnmt* on cardiac function, improving it in HSD and preventing its deterioration in this diet.
Of note, *Gnmt* overexpression in ND did not trigger cardiac dysfunctions (data not shown).
The results and conclusions have been corrected.

Interestingly, HSD itself tends to decrease AI, a further knockdown of GNMT further decreases AI. This indicates GNMT downregulation under HSD contributes to AI reduction. Together, GNMT downregulation under HSD prevents HP from going higher, while its downregulation causes AI going down.

In the manscript, the authors claim that " Gnmt KD led reduced HP and AI, suggesting that it is able to counteract the

effect of HSD observed in control flies on these phenotypes". This is not true according to the logic in Results section 1. As in section 1, the effect of HSD on AI is not significant, so the authors shouldn't say" HS tended to reduce AI".

Our reanalyzes and new results showed no *Gnmt* impact on AI, so these Figure panels were removed.

Why GNMT knockdown reduced FS under ND (Fig. S3C), while increasing FS under HSD (Fig. 3F)? If GNMT knockdown induces hypertrophy, I would expect it to increase FS.

Gnmt overexpression did not affect cardiac diameters in HSD, but it nevertheless led to an increased contractile efficacy compared to HSD controls (**Figure S3F**).

These new results strengthen the positive effect of *Gnmt* on cardiac function, preventing its deterioration in sugar diet. The text was modified accordingly.

High Fat Diet modulated CD36-scavenger receptor and Glut8 orthologues

In this revised manuscript, we present RT-qPCR validating the downregulation of *Snmp1* expression and the slight upregulation of *nebu*'s in the heart of 10days old HFD-fed females compared to ND-fed ones (**Figure S3B**).

HFD: Snmp1 gene is downregulated, however, both overexpression and knockdown of Snmp1 in ND induced some phenotypes.

Indeed, as mentioned in the revised manuscript (**page 6, lines 21-24**), in heart of females fed ND, both *Snmp1* knockdown (*Snmp1KK*) and overexpression (*Snmp1*^{WT}) showed a reduction of EDD and ESD (**Figure 3J; S3J**) but FS is increased accordingly only in *Snmp1KK.*
As notified in the text, both downregulation and overexpression of *Snmp1* led to side-phenotypes (**page 6, lines 24-28**): *Snmp1KK* exhibited abdominal fat increase (**Figure S3K**) and ostial cells seemed clearly malformed in *Snmp1*^{WT} (**Figure 3M**). This may explain why the heart shows the same type of functional impairment in both genotypes.
We now discussed the hypothesis that these similar cardiac dysfunctions may result from *Snmp1* being a regulator of organismal or cardiac lipid homeostasis. Indeed, increasing body fat content is deleterious as is increasing the import of fat in the cardiomyocytes. Finally, both affects cardiac cells' health and functioning.

HFD: nebu has a role in regulating cardiac function under ND.

HSD and HFD revealed the secretory function of the heart

They identified diet-regulated secreted proteins that are required for cardiac dysfunction.

Cardiac Fit expression impacted Cardiac performance.

The author used Hand-G4 to knock down Fit using KK and GD lines, KK line showed a reduction in HP (Fig. 5A), but not GD line (Fig. S5D). How did the author conclude that Fit is required for cardiac function? Also, with the positive data, the difference is too subtle.

We apologize and agree that the contradictory or inconsistent results obtained with the two RNAi lines were confusing. For this revised version, we first assess the effect of the two RNAi lines (KK and GD) on *fit* expression in the dissected hearts. RT-qPCR for KK line is presented in **Figure S5A**. GD line did not show a significant reduction of *fit* expression

when expressed in the heart with Hand>, which can explain the former results presented (not shown but data are available). So, we removed all results obtained with the GD line in this revised version.
To confirm the KK effects, we used *fit* KO allele (*fit*^{81}) and truncated version of *fit*, without its signal peptide (*fit*^{DeltaSP}), which has a dominant negative effect, both previously published and validated (Sun et al. 2017, Nat. Comm.). These two mutants were used to investigate the cardiac function of *fit* in our analysis. Results presented in **Figure 5** and **S5** confirm the phenotypes already observed with the KK line when expressed with Hand> in the heart and with Lsp2> in the fat body.
Our results validate the effect of *fit* decrease on rhythmicity and contractility, the reverse effects being consistently observed in *fit* overexpression. In conclusion, we are confident in the requirement of Fit in the regulation of cardiac performance.
These new data are now included in the results section "Cardiac Fit expression impacted Cardiac performance" (**pages 8-9**)

Referee cross-commenting

i agree with the experiments proposed by reviewer 2.

Reviewer #1 (Significance (Required)):

The study aims to examine the effect of diet on cardiac function.

The strength is that a lot of characterisations were done.

the weakness is the functional data regarding fit could not be validated in two different RNAis, thus the evidence is not strong to support the conclusions.

We again would like to thank the reviewer for her/his remarks and suggestions. She/He highlights the weakness of the first analysis and this was an important and constructive feedbacks for us. We strengthened our results by increasing samples, reanalyzing data and performing mandatory new experiments that are now included in this revised version.

Reviewer #2 (Evidence, reproducibility and clarity (Required)):

In this manuscript, Khamvongsa-Charbonnier et al. reported a RNA-seq analysis and RNA interference screening on high-fat and high-sugar-induced cardiomyopathy in Drosophila. The authors uncovered novel genes in 1C-metabolism, galactose metabolism, CD36-scavenger receptor and glucose transporter, as adaptative factors of cardiac function under high-fat and high-sugar treatment. The authors also identified a satiety hormone, Fit, as a cardiokine to control food intake and , expressed by dilp5 secretion. In summary, this study leverages the powerful genetic model Drosophila to uncover a number of new factors in regulating cardiac function under nutritional stresses and potentially offers new insights into molecular mechanisms underlying diet-related cardiac diseases. I have a few concerns, as listed below.

First, we would like to thank the reviewer for her/his comments and suggestions that deeply help us to improve the take-home messages of our manuscript. Following her/his recommendations and suggestions, we can now present a revised and stronger version of our manuscript.

1. Quantitative RT-PCR is required to validate the expression patterns of candidate genes identified from the RNAseq analysis.

RT-qPCR have been performed on hearts dissected from 10 days old females fed ND, HSD or HFD. *Gnmt*, *Sardh* and *Galk* validated downregulation are presented in **Figure S3A**, *Snmp1* downregulation and *nebu* upregulation (trend but non-significant) in **Figure S3B**, *fit* downregulation in **Figure S5A**.

2. The authors state that the dysregulated gene expression patterns reflect acute adaptation to HSD and HFD stresses. Most of the candidate genes in this study were downregulated upon HSD and HFD. However, it is recommended that overexpression of these genes, rather than knockdown, is needed to confirm whether the downregulation of these candidate genes upon stresses is an adaptative response.

We agree with the reviewer and followed her/his recommendation when tools were accessible for our analysis.

For example, HSD feeding induces the heart period. Knocking down Gnmt, specifically in the heart, under the HSD feeding changes can reduce the heart period. This evidence is insufficient to suggest the protective role of Gnmt under the HSD diet. Gnmt has already been downregulated under the HSD. Further knockdown of Gnmt, instead of returning the Gnmt expression to normal levels, to protect cardiac contractile performance complicates the model.

We thank the reviewer for her/his suggestion. We used UAS-*GnmtWT* (from FlyORF) to perform these experiments. As shown in (**Figure 3C-E; S3C,F**), knocking down *Gnmt* in the heart increased HP, EDD, ESD and CO. In the same Figure panels and in **Figure S3F**, we showed that overexpressing *Gnmt* with Hand> in HSD was sufficient to rescue some sugar induced phenotypes or to induce some, when compared to corresponding controls evaluated in the same experiments in ND and HSD. *Gnmt* overexpression in ND did not trigger cardiac dysfunctions (data not shown).
HP increase and CO decrease are rescued by *Gnmt* cardiac overexpression in HSD. Interestingly, the cardiac constriction induced by HSD is not rescued by *Gnmt* overexpression, but it is enough to increase FS and CO in sugar diet. These new results strengthen the positive effect of *Gnmt* on cardiac function, improving it in HSD and preventing its deterioration in this diet.
Sardh knockdown in ND, resulted in milder phenotypes but induced significant hypertrophy in ND as *Gnmt* does. No available tools allowed us to test its overexpression in HSD.
Nevertheless, as mentioned and discussed in the manuscript (**page 5, line 27-30; page 11, lines 11-14**), such protective role of muscular function and integrity has already been characterized in fly IFM in time-restricted feeding experiments for *Gnmt* and *Sardh* (Livelo et al., 2023, Nat.Comm.). Our experiments show that both genes encounter the same role in cardiac function upon nutritional stresses. The text was modified accordingly.

3. The authors suggest that the effect of nebu on heart contractility is not dependent on diet. However, based on the result from Figure 3O-P, the HFD treatment blocks the effect of nebu knockdown on heart contractility. The authors need to further explain these results and modify their conclusions accordingly.

We completely agree with the reviewer. We did not correctly analyze these results. We reanalyze our data, taking into account only the experiments of *nebu* knockdown that were performed in ND and in HFD concomitantly. Results are shown in **Figure 3O-P; S3L-N**.
As mentioned in the manuscript (**page 7, lines 3-8**), *nebu* knockdown led to identical HP decrease in both diets but its constrictive effect (reduction of heart diameters) in ND is abrogated by fat diet.
We modified the text accordingly in the results and discussion (**page 7, lines 8-11; page 12, lines 7-12**).

4. It is a bit confusing that knockdown of fit using Hand-Gal4 induced food intake, but knockdown of fit using tin-Gal4 or Dot-Gal4 did not significantly induce food intake (Fig 6A). The author did not provide any explanation of these results.

What is even more confusing is that overexpressing fit using Dot-Gal4 decreased food intake, but overexpressing fit using Hand-Gal4 or tin-Gal4 did not significantly decrease food intake (Fig 6B). Why was the strong food intake phenotype not observed using Hand-Gal4 in both experiments?

These confusing results lead to a question, which cell type is responsible for the production of cardiokine, Fit?

We apologize for the misleading results presented in the initial manuscript. We hope that our revised version will clarify Fit function regarding its remote impact.

Concerning the requirement of Fit function and the cell types that produces Fit, the results we obtained when evaluating cardiac performance strongly suggest that both cardiomyocytes and pericardial cells are important and recapitulate the effect of Hand> (**Figure 5A-C; S5G-H**).

In the case of food intake measurements, we now present results with newly performed food intake experiments for the Hand>*fit*^{WT} (**Figure 6D**). They show a significant reduction of food intake in this condition, corroborating the results obtained with Dot>. We add a clarification in the manuscript for this point (**page 10, lines 11-16**).

5. When testing the role of cardiac Fit in Dilp5 secretion, the authors subjected flies to starvation stress. However, the main focus of the present study is on HSD and HFD. The RNAseq analysis showed that Fit expression was downregulated by both HSD and HFD. Can the authors show that Dilp5 secretion is reduced by both HSD and HFD? Most importantly, can the authors test whether overexpression of cardiac Fit blocks HSD- or HFD-reduced Dilp5 secretion?

We understand the point raised by the reviewer. First of all, we wanted to correlate the measured impact on food intake, when manipulating *fit* expression in the heart, to the level of Dilp release, as it has been used and validated in (Sun et al. 2017, Nat. Comm.). In this purpose, we used the same approach and protocol and results are shown in **Figure 6 E-F**.
As mentioned by the reviewer, *fit* expression is downregulated in both HSD and HFD (which we confirmed by RT-qPCR in **Figure S5A**). As suggested by the reviewer, we performed Dilp5 immunostaining on CNS from females that were fed HSD of HFD for 10 days. Our results, in **Figure 6B** (left panels) and corresponding quantifications in **Figure 6C**, show that both diets strongly induce a decrease in Dilp5 amount in the IPCs and that it was not due to an altered *Dilp2*

or *Dilp5* expression in the CNS (**Figure S6A**). In this condition, overexpressing *fit*, which has a promoting effect on Dilp secretion (**Figure 6B**, right panels ND), may only have an additive effect. This is shown in **Figure 6B-C**.

Reviewer #2 (Significance (Required)):

In summary, this study leverages the powerful genetic model Drosophila to uncover a number of new factors in regulating cardiac function under nutritional stresses and potentially offers new insights into molecular mechanisms underlying diet-related cardiac diseases.

We again would like to thank the reviewer for her/his remarks and suggestions. Her/His important and constructive feedbacks helped us to improve and strengthen our study. Despite the weak points of the first version, she/he had supportive feedback and we deeply thank her/him. This revised version had improved results and analysis, thanks to the use of new genetic tools that strengthen this analysis.
